# Development and validation in Ecuador of the EPD Questionnaire, a diabetes‐specific patient‐reported experience and outcome measure: A mixed‐methods study

**DOI:** 10.1111/hex.13366

**Published:** 2021-09-28

**Authors:** Jimmy Martin‐Delgado, Aurora Mula, Mercedes Guilabert, Carlos Solís, Lorena Gómez, Gustavo Ramirez Amat, José Joaquin Mira, EPD Research Group

**Affiliations:** ^1^ Atenea Research Group Foundation for the Promotion of Health and Biomedical Research Alicante Spain; ^2^ Health Services and Policy Research Group University of Exeter Exeter UK; ^3^ Instituto de Investigación e Innovación en Salud Integral, Facultad de Ciencias Médicas Universidad Católica de Santiago de Guayaquil Guayaquil Ecuador; ^4^ Health Psychology Department Miguel Hernández University Elche Spain; ^5^ Endocrinology Service Hospital IEES Norte Los Ceibos Guayaquil Ecuador; ^6^ Directora Técnica de Área Centro de Salud No. 1 Centro Histórico Quito Ecuador; ^7^ Centro de Salud Hospital Pla Health District Alicante‐Sant Joan Alicante Spain

**Keywords:** diabetes, mixed‐methods study, patient‐reported experience measures, patient‐reported outcome measures, questionnaire, vulnerable populations

## Abstract

**Introduction:**

The global prevalence of diabetes in 2019 in adults was estimated to be 9.3%. This study developed in Ecuador, for the first time, instruments to assess patient‐reported outcomes and experiences.

**Methods:**

The Experiences of the Person with Diabetes (EPD) Questionnaire is a diabetes‐specific instrument. A mixed‐methods study was conducted. First, a qualitative item development phase that included four focus groups and six semi‐structured interviews with patients was conducted in different rural and urban areas of Ecuador to obtain information on culture, beliefs, demographics, diet and social perspectives. A second quantitative phase for psychometric validation was carried out in primary care settings of rural and urban areas of Ecuador.

**Results:**

Forty‐two and four hundred and eighty‐nine participants were included in each phase, respectively. The item development phase resulted in a questionnaire of 44 items (23 for perceived outcomes and 21 for experiences). In the validation study, most participants were women (58%) and from urban areas (57%). Exploratory factor analysis revealed three dimensions for each instrument. Outcomes instrument dimensions were symptoms and burnout, worries and fears and social limitations. Experiences instrument dimensions were information, patient‐centred care and care delivery. Cronbach's *α* values of the total score and dimensions were high, ranging between .81 and .93 in both instruments. Confirmatory factor analysis showed an acceptable fit of the data.

**Conclusion:**

The EPD Questionnaire is probably the first instrument developed to assess patient‐reported experiences and perceived outcomes in a middle‐income country that included patients to capture all dimensions relevant for the intended population. Its psychometric properties are robust and could provide valuable information for clinicians and policymakers in the region.

**Patient or Public Contribution:**

The development of these instruments has taken into consideration patients and the public since their conception. A qualitative approach gathered relevant information related to the cultural, social and economic burden of different populations in Ecuador. Before validation, a pilot test was carried out with users of the National Health Services to obtain their perspectives and insights of the developed instrument. Finally, during the data analysis, we have given special consideration to social variables such as rural and urban populations.

## 1 INTRODUCTION

Chronic noncommunicable diseases are a challenge for all health systems. The global prevalence of diabetes in 2019 in adults (20–79 years) was estimated to be 9.3% (463 million people), expected to increase to 10.2% (578 million) by 2030.^1^ However, more alarmingly, the prevalence of diabetes has increased more rapidly in low‐ and middle‐income countries^2^ with health systems where much remains to be done. It is estimated that by 2030, in America, 83 million people may have diabetes, an increase of 50% since 2000.^3^ In Ecuador, the prevalence of diabetes is 7.3%.^4^, ^5^

In Ecuador, diabetes is the second most common cause of death, only after ischaemic heart disease,^6^ which is also a well‐established risk factor. Driven by obesity (6 out of 10 Ecuadorians are overweight and obese), unhealthy lifestyles (60% have a sedentary lifestyle) and increased life expectancy (74 years for men and 80 years for women), diabetes carries a high burden of disease, derived from its prevalence, complications and the associated comorbidity.^4^, ^7^ Ecuador's Health System is struggling to achieve all its objectives. Despite the significant increase in the number of medical consultations, this effort has not been reflected in a real impact in terms of health improvements for the population.^8^ One of the strategies available in primary care is peer‐related support groups such as the Club of Patients with Chronic diseases, led by a healthcare worker (medical doctor or nurse). These Clubs of Patients have been expanded in the country, aimed at providing education and counselling to patients with chronic diseases, especially diabetes and hypertension.^9^

The prevention and control of diabetes represent a challenge for professionals, healthcare services and social systems and, above all, for the patients themselves, since diabetes significantly affects their quality of life, requiring a coordinated effort from different levels of care to alleviate its effects. In addition, diabetes has a high economic and social impact.^10^ The promotion of a healthy lifestyle, control of risk factors (diet, overweight, physical exercise), diabetes education and patient self‐care are essential elements in preventing the progress of the disease and the social and health overload that it represents.^11^

For a better intervention in terms of health–disease processes, the patient with a chronic disease has to be an active protagonist.^12^, ^13^ Healthcare systems aiming to achieve person‐centred coordinated care should systematically assess patients' perspectives.^14^ A recent scoping review of patient‐reported measures could not retrieve any in situ developed diabetes‐specific tool in low‐ and middle‐income countries.^15^ All of them were developed in high‐income countries with strong economies and robust healthcare systems. It is essential to incorporate the patient's voice in a healthcare organisation to obtain feedback and improve patient participation.^16^

In low‐ and middle‐income countries, out‐of‐pocket expenditure accounts for 48% and 31% of healthcare financing, respectively.^17^ Specifically in Ecuador, out‐of‐pocket payments account for 40% of healthcare expenditure,^18^ combined with difficult access to health services, especially in more distant communities; the economic burden is a common issue in the local context.^19^, ^20^ Although there is local production of first‐hand supplies, the social and economic difficulties, especially among vulnerable populations, make them more likely to consume high‐carbohydrate diets due to their lower cost. Other cultural and educational factors result in people holding diverse beliefs about the pathogenesis of the disease, which complicates health education and management. Therefore, there might be a current need to develop diabetes‐specific instruments in low‐ and middle‐income countries, where social, cultural and economic contexts and access to healthcare are different.

The Triple Aim is to improve individual experiences, the health of populations and reduce per capita costs of care of populations.^21^ Healthcare systems aiming to achieve person‐centred coordinated care should be organized to support value‐based healthcare centred on what matters most to patients. The incorporation of instruments known as patient‐reported experience measures (PREMs) has made possible to make the patient visible within the context of health systems and include his or her experience in managing chronic pathologies. One of the most widely used relational PREMs is the Picker Patient Experience Questionnaire.^22^ CARE measure^23^ and IEXPAC^24^ are clear examples of a functional PREM. The results are better when patients and professionals agree on therapeutic alternatives and make joint decisions, improving their satisfaction and clinical results^25^, ^26^ measurable through patient‐reported outcome measures (PROMs).^27^, ^28^ The adoption of these measures by healthcare systems would reduce overuse of care. Among the most widely used PROMs for measuring the quality of life of patients with diabetes are the ADDQoL,^29^ the WHO Well‐Being questionnaire and the EuroQoL‐5D. However, two of these three are not specific to diabetes.

Nowadays, healthcare services should not be evaluated without patients' perspectives, taking advantage of the information that they provide to increase quality levels of healthcare services.^30^ Available instruments may have limitations, as mentioned before. The objective of this study was to develop in Ecuador, for the first time, PREM and PROM instruments that are adapted to local needs and contexts and, second, to determine the experience of people with diabetes about ‘What is important for me about living with diabetes’?

## 2 MATERIALS AND METHODS

**Figure 1:**
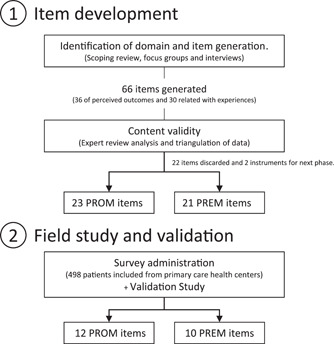
Flowchart of the study. PREM, patient‐reported experience measure; PROM, patient‐reported outcome measure

The development of the ‘Experiencias de la Persona con Diabetes*’* (EPD) Questionnaire, which included an independent PREM and PROM instrument, followed a mixed‐methods study. The protocol of this study considered the three phases used to create a scale described by Boateng et al.^31^ (item development, scale development and scale evaluation), the standards and guidelines for validation practices summarized by Chan^32^ and the COSMIN recommendations.^33^ Ethics approval was obtained for both item development and validation phases from a local ethics committee (HCK‐CEISH‐19‐0041) (Figure 1).

### 2.1 Item development

First, a scoping review of existing specific type 1 and 2 diabetes patient‐reported outcome and experience measures was considered.^15^ In a previous study, dimensions assessed by other measures, possible items and existing gaps were identified.

Second, four focus groups and six semi‐structured interviews involving patients and professionals were conducted in Ecuador between May and August 2019. To obtain information on the culture, beliefs, demographics, diet, type of treatment and degree of engagement, participants from the highlands, coastal and urban or rural areas were included.

Focus groups were conducted in four different locations in Ecuador, Quito, the capital city located in the highlands, Guayaquil, the largest coastal city, Mulliquindil, a rural highland area 123 km south of Quito and Campozano, a rural coastal area 117 km northwest of Guayaquil. Each focus group lasted between 45 and 60 min. The six semi‐structured interviews took place in a basic hospital in Guayaquil, lasting for around 40 min each. A purposive sampling strategy^34^ was used to recruit individuals with type 2 diabetes who might be interested in discussing their life experience.

Participants were recruited using a snowball approach and reached through phone calls. Direct conversations were held with the medical staff or the Club of Patients with Diabetes (a peer‐related support group) of the healthcare centre. Eligibility criteria were adults over the age of 18 years with type 2 diabetes. Once they had been invited, participants were explained the aim of the focus groups and they decided whether to participate voluntarily. Informed consents were signed before the focus groups were conducted, and permission for audio recording was obtained. They were also informed that they had the right to leave the focus group at any time without providing a reason. None of the participants declined the invitation to be part of focus groups or interviews. Data collection was continued until data saturation.

The interview guide content was developed through the mentioned scoping review on diabetes^15^ and was agreed by the team in consultation with professionals based in Ecuador (File S1). Experts in the qualitative research methodology provided input on the clinical approach and guidance. The focus groups were conducted in private rooms in the same health centres where participants used to receive medical attention to provide a familiar environment to generate trust. None of the healthcare workers were part of the research team or were involved during data acquisition. Participants' anonymity and data confidentiality were always respected. The focus groups began with a brief description of the aim of this study and a brief introduction of each participant, after informed consent was obtained. Afterwards, the moderator began with an open question asking participants to describe their experiences living with diabetes, important aspects concerning initial diagnosis, follow‐up (treatment and symptoms), barriers to a fulfilling life with diabetes, their main concerns and social and work life.

### 2.2 Dimensions explored for measure development

Data from all interviews and focus groups were transcribed and coded for emergent theme analysis to identify the common dimensions and concepts providing an underlying theoretical structure. The dimensions considered for analysis focused on the specific characteristics of the local context of the social, cultural and economic factors and the healthcare system. The data were then analysed by emergent theme analysis by two of the authors. Items were generated for each of the identified dimensions with an iterative process using deductive methods on the basis of the previously conducted scoping review of measures^15^ and inductive methods on the basis of the results of qualitative analysis and the experience and input of the research group.

### 2.3 Item scoring

The EPD Questionnaire included two instruments. The recall period was last month, and responses were rated on a four‐point Likert scale (1 = *hardly ever* to 4 = *everyday*). Both types of response scales, continuous rating and Likert‐type scales, are usually used and were tested to obtain patients' points of view. Patients chose the Likert‐type scale as easier to answer. Additionally, the intermediate point was removed, facilitating the interpretation and decision‐making of the participants. Scores were calculated as the sum of individual scores for each scale. Higher scores indicate better outcome results or better experiences with the healthcare care services provided. For the PROM scale, scoring was inversed (4 = *hardly ever* to 1 = *everyday*).

### 2.4 Content and face validity

To determine the ease with which patients answered the EPD Questionnaire, face validity, content and feasibility were assessed. A pilot test with type 2 diabetes patients was performed to evaluate if any other theme was missing. Also, wording, completion time, importance, difficulty of responding to each item and if items addressed their experience and outcomes adequately were included. A 5‐point Likert scale was used (0 = *it is not understood* to 5 = *completely understood*) and if items addressed their experience and outcomes adequately. Finally, they were asked if the response scale was appropriate for the instrument. Ten Participants were reached by their family doctor and had to be of legal age, diagnosed at least 5 years ago with type 2 diabetes and regular users of health services (patients for at least 1 year).

### 2.5 Reliability and validity study

A paper‐based validation study was conducted during January and March 2020 to collect data to assess the measurement and psychometric properties of the EPD Questionnaire. The study was conducted in the primary care settings of rural and urban areas of Ecuador. To be eligible for the study, participants had to be of legal age, been diagnosed with type 2 diabetes and be able to understand the questions. The sample size was established using the formula for finite universes considering the last available prevalence data of diabetes in Ecuador.^5^ A 95% confidence level, 5% accuracy (*p* = *q* = 50) and 15% of lost data were established, and the criteria to involve at least 10 respondents per item^31^ were applied. The selection process of the respondents was randomly performed by healthcare professionals working in each of the participating health centres.

### 2.6 Item reduction

Exploratory principal component factor analysis (EFA) was used to evaluate the scales' latent content structure. In this process, the varimax rotation was used, as it usually produces explicit results that can facilitate the interpretation. Furthermore, items with cross‐loadings and factor loadings of less than 0.50 were dismissed using IBM SPSS Statistics for Windows, Version 25.0. Items were also reviewed by the research team composed of medical doctors, psychologists and experts in mixed‐methods research and patients.^35^

### 2.7 Reliability

Internal consistency was measured using Cronbach's *α* and McDonald's *ω*. A minimum correlation of .70 was expected to establish that the instrument is internally consistent and has acceptable reliability. The split‐halves method was used as an alternative to test–retest. Split‐halves provide different results; we have considered Guttman's *λ*_4_ as it provides the most significant split‐half reliability for measures with 16 items or less.^36^ Items were randomly and evenly distributed using the R package splitHalf.

### 2.8 Construct validity

Confirmatory factor analysis (CFA) was used to confirm the underlying theoretical structure, estimating several fit indices to test which CFA model best represented the data set: the CFI—Comparative Fix Index; AGFI—Jöreskog‐Sörbom Fit Index–Goodness of Fix Index; SRMR—Standardized Root Mean Square Residual; and GFI—Jöreskog‐Sörbom Fit Index Goodness of Fix Index. The R package Lavaan was used.^37^ We calculated the Pearson correlation coefficients between all items of the three factors of the PREM and PROM instruments and between each dimension and total score. This enabled us to evaluate the convergent and discriminant validity based on the hypothesis that the correlations between each item would be stronger than those between factors and the total score.

### 2.9 Structural validity

Additionally, the hypothesis that scores on the scale would be higher in urban areas compared to rural areas was tested. This is because in urban areas, patients have higher education levels and improved access to health. The Mann–Whitney *U* test was used.

### 2.10 Responsiveness

To assess responsiveness, in both instruments, we evaluated how patients' responses changed according to years from diagnosis, hypothesising that scores in the experiences measure would be better in patients with a longer history of diabetes. Also, scores in the perceived outcomes measure, especially the dimension of fears and social limitations, could worsen over time. The Kruskal–Wallis test was used.

### 2.11 Minimal clinically important change

The minimal clinically important change (MCIC) was estimated using three distribution‐based approaches: 1 standard error of measurement, 0.5 standardized effect size and 0.5 responsiveness statistic.^38^, ^39^ The values enable estimation of the amount of change for each of the scales to be considered important.

## 3 RESULTS

### 3.1 Item development

A total of 28 participants of the focus groups were reached through their medical doctors who were directly involved with their care. Afterwards, 36 phone calls were made, and 14 more patients accepted the invitation (38%) to participate in the focus groups. Data saturation was achieved with the third focus group and six interviews, but the fourth focus group was conducted to comply with the methods of including a focus group from a rural coastal area. Detailed characteristics of the participants are presented in Table 1.

**Table 1:** Characteristics of participants involved in the qualitative research phase

	Urban area (*N* = 23)		Rural area (*N* = 19)	
	Coast	Highlands	Coast	Highlands
Men/women	3/11	1/8	1/8	5/5
Age	Mean: 53.2 (33–75)	Mean: 54.8 (49–63)	Mean: 66.2 (44–73)	Mean: 54.2 (47–64)
No pharmacological treatment	1	0	0	0
Oral antidiabetic drugs	5	6	9	10
Combined treatment	5	3	0	0
Actively involved in Club of Patients with Diabetes	2	7	0	5

Among the most prevalent symptoms, thirst was described as a persistent cause of discomfort, along with fatigue.

*Even if I drank water, I was thirsty. I was even more thirsty at night and dawn and used the bathroom a lot*.

A proportion of the participants accepted their pathology, but not the treatment. This is motivated by a high prevalence of alternative treatments, lack of information, the low level of health literacy and ‘fear’ of insulin therapy.

*I had a hard time accepting that I have diabetes*.

*The doctors told me not to stop taking insulin, but I stopped anyway*.

*I do not take medicines; I drink natural waters*.

No clinical therapeutic targets agreed upon with the patients were established. Among the main fears are the long‐term complications (diabetic nephropathy and retinopathy) since this would adversely affect the autonomy they maintain and limit them from leading a ‘normal life’. A complete list of verbatims is included as File S2.

*My fear was to go blind because you see people who go blind (…) being disabled is worrying*.

*What worries me most is getting to dialysis, but there are times when I forget about it*.

By means of the scoping review^15^ and qualitative data analysis, a total of 66 items were generated, 36 related to outcomes and 30 related to experiences during the item development phase. For each of the proposed categories, a minimum of three items was generated. Following expert review analysis and cross‐tabulation of verbatims and items, 22 items were discarded. Finally, a total of 44 items were included for the evaluation of content and face validity. These 44 items were divided into two domains of outcome and experiences. The EPD Questionnaire had two instruments, a PROM with the following dimensions of symptoms and burnout, worries and fears and social limitations, and 23 items. The PREM dimensions included information, patient‐centred care, care delivery and care planning, and 21 items.

### 3.2 Content and face validity

A total of 10 participants with type 2 diabetes, seven women and three men, with a mean age of 67.4 years, and 7.1 years from the diagnosis of diabetes. Respondents understood approximately 90% of the items. Two questions were rephrased following recommendations by participants to improve comprehension (I use natural treatments instead of pills, and I felt that the doctor listened to me in the consultation). Participants were also given the opportunity to choose between two different 4‐point Likert scale responses; 9 out of 10 picked the following scale (*hardly ever* to *everyday*).

### 3.3 Reliability and validation study

A total of 498 participants from five different locations (rural, urban, coastal and highlands) in Ecuador responded. After excluding lost data, the remaining 486 valid questionnaires were included for analysis. The recall period of the questionnaire was the last month (this is because follow‐up consultations are monthly), and the time taken to fill the questionnaire was 5 min. The mean age of the participants was 59.2 years (confidence interval [CI] 95%: 57.9–60.5). The mean age from the diagnose of diabetes and treatment was 8.9 (CI 95%: 8.1–9.7) and 8 (CI 95%: 7.4–8.6), respectively. A total of 11 (2.3%) patients were hospitalized in the last year due to diabetes, with a mean of 9.5 (8–11) days of hospital stay. Table 2 shows the detailed characteristics of the participants.

**Table 2:** Characteristics of the participants involved in the validation study

	*N*	%
Sex		
Men	205	42.0
Women	283	58.0
Town		
Rural	207	42.4
Urban	281	57.6
Age (years)		
18–64	296	60.7
65–74	121	24.8
≥75	71	14.5
Years from diagnosis		
≤1	66	13.5
2–10	271	55.5
11–20	108	22.1
≥21	35	7.2
BMI		
<25	124	25.4
25–29.99	149	30.5
30–39.99	168	34.4
≥40	34	7.0
Arterial hypertension		
No	225	46.1
Yes	218	44.7
Use of natural treatments		
No	430	88.1
Yes	53	10.9
Years in treatment		
≤1	72	14.8
2–10	281	57.6
11–20	112	23.0
≥21	20	4.1

Abbreviation: BMI, body mass index.

### 3.4 Item reduction

The EFA identified five factors for the PROM and three factors for the PREM, explaining 70% and 62% of the variance, respectively. Items that did not show factor loadings of 0.50 or higher were removed. This process was repeated on two occasions, and 19 items were excluded. Furthermore, the remaining items were assessed for content redundancy. Following this approach, four items with similar content and connotation were discarded. In each case, the item with higher factor loading was retained. One item related to insulin intake was removed due to a low response rate.

**Figure 2:**
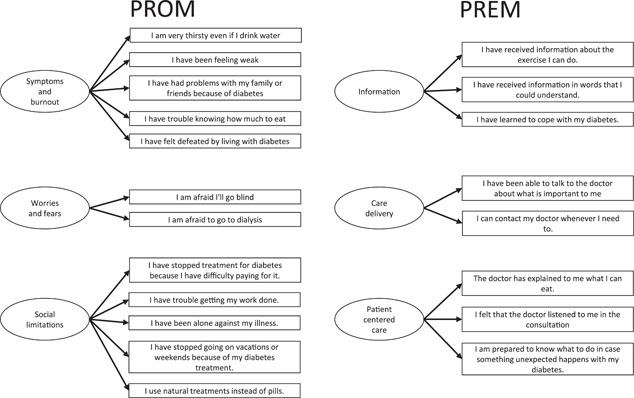
Patient‐reported outcome measure (PROM) and patient‐reported experience measure (PREM) structure for validation

Finally, EFA suggested a three‐factor structure for both instruments. The PROM explained 72.5% of the variance, and the PREM explained 87.06% of the variance. The varimax rotation converged after five iterations in both cases. Tables 3 and 4 show the rotated factor loadings for each of the instruments. According to factor loadings, the PROM instrument had 12 items and measured symptoms and burnout (1, 2, 8, 11, 12), worries and fears (Items 3, 4) and social limitations (5, 6, 7, 9, 10). The PREM instrument had eight items and measured the following dimensions: information (Items 3, 5, 6), patient‐centred care (Items 2, 4, 7) and care delivery (Items 1, 8). Figure 2 shows the dimensions and items of each of the instruments.

**Table 3:** Rotated factor loadings of the patient‐reported experience measure

	Factor 1	Factor 2	Factor 3
5. I have received information in words that I could understand	0.90		
6. I have learned to cope with my diabetes	0.88		
3. I have received information about the exercise I can do	0.88		
7. I am prepared to know what to do in case something unexpected happens with my diabetes		0.88	
2. The doctor has explained to me what I can eat		0.87	
4. I felt that the doctor listened to me in the consultation		0.86	
8. I can contact my doctor whenever I need to			0.94
1. I have been able to talk to the doctor about what is important to me			0.89

*Note*: Extraction method: Principal component analysis.

Rotation method: Varimax with Kaiser normalisation.

**Table 4:** Rotated factor loadings of the patient‐reported outcomes measure

	Factor 1	Factor 2	Factor 3
12. I have felt defeated by living with diabetes	0.86		
11. I have trouble knowing how much to eat	0.85		
8. I have had problems with my family or friends because of diabetes (e.g., an argument about what I can eat)	0.80		
1. I am very thirsty even if I drink water	0.76		
2. I have been feeling weak	0.75		
5. I have stopped treatment for diabetes because I have difficulty paying for it		0.86	
6. I have trouble getting my work done		0.85	
9. I have stopped going on vacations or weekends because of my diabetes treatment		0.82	
10. I use natural treatments instead of pills		0.81	
7. I have been alone in the face of illness		0.76	
3. I am afraid I'll go blind			0.93
4. I am afraid to go to dialysis			0.79

*Note*: Extraction method: Principal component analysis.

Rotation method: Varimax with Kaiser normalisation.

### 3.5 Reliability

Internal consistency coefficients measured by the Cronbach's *α* of the EPD Questionnaire (total score and dimensions) were high, ranging between .81 and .93 (Table 5). Split‐halves reliability values measured by Guttman's *λ*_4_ were 0.93 and 0.95 for PROM and PREM, respectively.

**Table 5:** Internal consistency of the EPD Questionnaire

Scale identification	Items	Min–max	Mean (95% IC)	SD	*α* Coefficient	McDonald's *ω*
Total PREM	8	8–32	19.3 (18.8–19.8)	5.6	.87	0.95
Factor 1: Information	3	3–12	6.4 (6.1–6.6)	2.8	.93	0.93
Factor 2: Patient‐centred care	3	3–12	7.5 (7.3–7.7)	2.6	.92	0.92
Factor 3: Care delivery	2	2–8	5.4 (5.2–5.6)	1.8	.83	0.83
Total PROM	12	12–48	39.2 (38.8–39.7)	5.0	.83	0.91
Factor 1: Symptoms and burnout	5	5–20	16.6 (16.4–16.9)	2.8	.88	0.89
Factor 2: Worries and fears	2	2–8	5.4 (5.2–5.6)	1.9	.81	0.81
Factor 3: Social limitations	5	5–20	17.2 (17.0–17.4)	2.5	.88	0.88

Abbreviations: CI, confidence interval; EPD, Experiencias de la Persona con Diabetes; PREM, patient‐reported experience measure; PROM, patient‐reported outcome measure.

### 3.6 Construct validity

To test the observed factor structure, CFA was used (Table 6). CFA showed an acceptable fit of the data for both patient‐reported outcomes and experience measures. File S3 shows the EPD Questionnaire with both PREM and PROM instruments.

**Table 6:** Confirmatory factor analysis of the EPD Questionnaire

Goodness‐of‐fit index	PREM	PROM
CFI	0.979	0.930
AGFI	0.913	0.850
SRMR	0.024	0.062
GFI	0.956	0.902
RMSEA	0.049	0.057
IC 90% RMSEA	0.036–0.063	0.050–0.065

Abbreviations: AGFI, adjusted goodness‐of‐fit index; CFI, comparative fit index; EPD, Experiencias de la Persona con Diabetes; GFI, goodness‐of‐fit index; PREM, patient‐reported experience measure; PROM, patient‐reported outcome measure; RMSEA, root mean square error of approximation; SRMR, standardized root mean square residual.

Convergent and discriminant validity analyses showed that PREM items adequately converge (>0.70) and factors discriminate each other (<0.23). Similar results were obtained for the PROM instrument items converge (>0.50), and factors discriminate each other (<0.21).

### 3.7 Structural validity

Overall, the PREM and PROM results were better in urban rather than rural areas (Tables 7 and 8). This might be due to overall access to health and information. Persons in urban areas had better results related to patient‐centred care and care planning (*p* < .05).

**Table 7:** Differences in patient‐reported experiences according to location

	Rural (*N* = 207)	Urban (*N* = 281)	*p*‐Value
1. I have been able to talk to the doctor about what is important to me	2.7 ± 1.0	2.9 ± 1.0	.02
2. The doctor has explained to me what I can eat	2.4 ± 0.9	2.6 ± 1.0	.02
3. I have received information about the exercise I can do	2.0 ± 0.9	2.2 ± 1.1	.07
4. I felt that the doctor listened to me in the consultation	2.4 ± 0.9	2.6 ± 1.0	.007
5. I have received information in words that I could understand	2.1 ± 0.9	2.3 ± 1.1	.2
6. I have learned to cope with my diabetes	2.0 ± 0.9	2.2 ± 1.0	.2
7. I am prepared to know what to do in case something unexpected happens with my diabetes	2.4 ± 0.8	2.5 ± 1.0	.3
8. I can contact my doctor whenever I need to	2.5 ± 0.9	2.7 ± 1.0	.02
Factor 1: 3, 5, 6	6.1 ± 2.3	6.6 ± 3.2	.4
Factor 2: 2, 4, 7	7.2 ± 2.3	7.7 ± 2.8	.02
Factor 3: 1, 8	5.2 ± 1.7	5.6 ± 1.8	.007
Total	18.4 ± 4.4	19.8 ± 6.2	.1

**Table 8:** Differences in patient‐reported outcomes according to location

	Rural (*N* = 207)	Urban (*N* = 281)	*p*‐Value
1. I am very thirsty even if I drink water	3.0 ± 0.9	3.4 ± 0.7	<.001
2. I have been feeling weak	3.2 ± 0.7	3.4 ± 0.7	.001
3. I am afraid I'll go blind	2.3 ± 1.1	2.7 ± 1.0	<.001
4. I am afraid to go to dialysis	2.6 ± 1.1	3.0 ± 1.0	<.001
5. I have stopped treatment for diabetes because I have difficulty paying for it	3.4 ± 0.6	3.5 ± 0.6	.04
6. I have trouble getting my work done	3.4 ± 0.6	3.5 ± 0.6	.04
7. I have been alone in the face of illness	3.3 ± 0.7	3.4 ± 0.6	.02
8. I have had problems with my family or friends because of diabetes (e.g., an argument about what I can eat)	3.3 ± 0.6	3.4 ± 0.6	.004
9. I have stopped going on vacations or weekends because of my diabetes treatment	3.4 ± 0.7	3.5 ± 0.6	.1
10. I use natural treatments instead of pills	3.4 ± 0.6	3.5 ± 0.6	.3
11. I have trouble knowing how much to eat	3.2 ± 0.7	3.4 ± 0.7	<.001
12. I have felt defeated by living with diabetes	3.3 ± 0.7	3.4 ± 0.6	.002
Factor 1: 1, 2, 8, 11, 12	15.9 ± 2.8	17.1 ± 2.7	<.001
Factor 2: 3, 4	5.0 ± 2.0	5.7 ± 1.8	<.001
Factor 3: 5, 6, 7, 9, 10	16.9 ± 2.4	17.4 ± 2.5	.02
Total	37.8 ± 4.6	40.2 ± 4.9	<.001

### 3.8 Responsiveness

Experiences of persons with diabetes improved over time, with better results in factors related to information, patient‐centred care and care planning (*p* < .05), even though problems related to care delivery persist. Outcomes related to worries and fear worsen over time: 6.2 for persons with a recent diagnosis instead of 3.6 in persons who have had the diagnosis for 20 years or more (*p* < .05). File S4 includes detailed information on each item and factor.

### 3.9 Minimal clinical important change

The MCIC ranged from 0.74 to 1.6 points for the information factor, from 0.73 to 1.4 points for patient‐centred care and from 0.74 to 0.9 points for care delivery. For the PROM, the ranges of MCIC were 0.97–1.4 points for the symptoms and burnout factor, 0.83–1 for worries and fears and 0.87–1.3 for social limitations (File S5).

## 4 DISCUSSION

Disease‐specific patient‐reported measures are crucial for assessing treatment benefits, tailoring treatment to patients' needs and personal context and providing valuable data for policymakers and health systems. The EPD Questionnaire is a diabetes‐specific instrument developed according to current standards and expert consensus,^31^, ^32^, ^33^ and more importantly, the first PROM and PREM instrument to be fully developed in Ecuador, a middle‐income country. The developed measures have considered the social, cultural, economic and healthcare needs of the local context. An example of this is the inclusion of a specific question related to the regular use of natural treatments, not available in other instruments. This instrument has been found to have adequate validity and reliability and should provide accurate and targeted information for local policymakers and the region.

The United Nations Sustainable Developments goals^40^ aim to promote healthy lifestyles and well‐being. These strategies seek to empower citizens and patients to make decisions regarding their health. It remains a challenge for local health authorities in South American countries and the Pan American Health Organization to put patients at the centre of care. This questionnaire aims to address this challenge and can improve the patient‐centred care approach in Ecuador by measuring the results of care from the patients' perspectives and expectations.

It is fundamental to achieve person‐centred care and to do, so individuals need to be part of the healthcare system and process. Patient‐centred care and cocreation of care were associated positively with satisfaction with care and the physical and social well‐being of patients with multimorbidity in the primary care setting.^41^ The development of this instrument has included the participation of patients since its beginning. A qualitative study was performed in different social, cultural and geographical areas of Ecuador. This approach had the objective of capturing patients' beliefs, values, way of living and how they experience ‘living with diabetes,’ as these could influence results.^42^, ^43^ The results of the validation study suggest that the EPD Questionnaire is psychometrically sound. The PREM explained 87% and the PROM explained 72.5% of the variance, and correlations indicate a strong relationship between items and internal consistency with its respective dimension. Fit indices show that the final version of both instruments' outcomes and experiences addresses the core constructs (experiences constructs of information, patient‐centred care and care delivery and outcomes constructs of symptoms and burnout, worries and fears and social limitations) that they intended to measure. Ecuador is currently reviewing its diabetes care programme at the time of this study. The indicators currently proposed to measure effectiveness and efficiency do not include the patients' points of view, both in terms of their experience throughout the care process and the outcomes achieved. The EPD Questionnaire could help fill this gap.

PROMs usually focus on a healthy lifestyle, diabetes‐related distress and social support. In this outcome and experience instrument, we have included other aspects relevant for patients.^24^ For an instance, if individuals received integrated health and social care when required, how their occupational or leisure activities with friends and family are affected due to their diabetes, use of natural treatments, if the information provided was easy to understand, access to healthcare and economic burden, of special relevance because in middle‐income countries out of pocket payments represents 30% of the health expenditure.^17^ All of these are of particular interest when tailoring an instrument to the intended population.^44^ Most patient‐reported measures have been developed in high‐income countries, where healthcare systems are more robust and capable of achieving better results. These other instruments have taken into account other dimensions and outcome variables such as e‐health, device functionality, insulin pumps or pens and individualized therapeutic plans^24^, ^45^, ^46^ that may not apply to all contexts, due to several reasons such as lack of internet access or national system coverage of insulin pumps. In our study, we have assessed results taking into consideration urban and rural areas. Each of them presents different characteristics for individuals, related to access to health, income and education.^47^ In general, individuals in urban areas show better results in PREM and PROM, indicating a flaw of the national health system.

During this study, some of the weaknesses regarding the care process for persons with diabetes have become indirectly visible, such as the difficult access to healthcare in rural populations, the lack of medicines and supplies and limited access to laboratory tests (HbA1c). The lack of flexibility of the Ecuadorian health system^8^ is another obstacle towards achievement of person‐centred care. Other challenges include differentiated measures for the indigenous population with different values and beliefs (cosmovision). The indigenous cosmovision interprets life in plenitude as a harmonious realisation between man and nature. In Ecuador, the cultural heritage of native populations includes the concept of Sumak or Alli, which means life in plenitude or well‐being.^48^ This belief modifies the more scientific perspective about the causes and approaches to diabetes and other chronic pathologies. Nevertheless, at the same time, local authorities should involve community leaders who can encourage peer support and strengthen support groups for patients who are already part of a health system and accustomed to following procedures.

This study reflects the convenience of combining the results of these instruments that include the patients' perspectives with other sources of objective evaluations of health outcomes such as glycosylated haemoglobin or population‐based indicators related to chronic diseases. However, special attention must be paid to address the needs of rural populations or those who live far from the communities where health centres are located.^49^ These instruments could also be used in other Latin American countries that share with Ecuador similar characteristics described previously. For an instance, coastal and andean population, similar customs and beliefs, and a health system where many remains to be done. At least, it can be assumed that this instrument is closer to the reality experienced in these countries than others originally designed and validated in developed countries, with more robust health systems, different beliefs and customs and primarily English‐speaking populations.

Furthermore, using these types of instruments in health policy decision‐making and incorporating patient perspectives, health planning decisions are legitimized, attention is brought closer to the needs identified by patients and improvements can be made to meet these needs.^50^ Moreover, it is expected that health outcomes improve when the patient becomes more involved and participates more actively in his or her self‐care.^51^ At a time when, as a result of the COVID‐19 pandemic, many of the consultations with patients have been interrupted, empowered patients have become more capable of taking care of their health in the absence of traditional consultation resources. The national health system in Ecuador has tried to adopt telemedicine, but only 44.5% of users reported a positive experience during 2020.^52^ This situation has drawn attention to the downsides of patients being dependent on healthcare providers and not actively assuming a self‐care role.^53^ In the current process of reviewing the diabetes care programme, it is an opportunity to include, to some extent, the voice of the patients with an in situ developed instrument to systematically measure and tackle some of the current challenges (access to healthcare, urban and rural disparities, economic burden, health beliefs and patient education and empowerment).

We encountered some limitations during our study. The use of HbA1c to compare scores and assess predictive validity was not possible due to intrinsic difficulties such as access to health or lack of standardized laboratory procedures. After the national lockdown was decreed on 17th March due to the COVID‐19 pandemic, the study had to be stopped, and test–retest assessment could not be performed. Another cross‐culturally validated diabetes‐specific instrument in the local context is not available, which did not allow comparison of our results with another measure. Additionally, we are aware of the limitations of a two‐item factor, but this was retained to have a short instrument. The association of PROMs and PREMS with HbA1C is likely to be complex. Future research should include standardized HbA1c testing and validation of another measure for anchor‐based MCIC and comparison with other scales.

## 5 CONCLUSION

In summary, we believe that the EPD Questionnaire is the first PREM and PROM instrument developed in a middle‐income country that included a mixed‐methods study to capture all dimensions relevant for the intended population. Its psychometric properties are robust and could provide valuable information for clinicians, policymakers and middle management in Ecuador and other countries of South America. Further research is needed to assess predictive validity with a more objective outcome such as HbA1c.

## THE EPD RESEARCH GROUP

The EPD Research Group comprised of Andrea Aguilar‐Aviles, María Castro García, Yamil Cornejo Guerrero, Sandra Gonzalez Chamorro, Lucia Gutierrez Dávila, Karla Mayorga Alvarado, Doménica Mendoza Macías, Carla Mina Curay, Carmita Pérez López, Martha Quijije, Katterin Romero Bello, Daniel Sornoza Arias, Eric Urquizo Rodriguez, Richard Vaca Maridueña, Jenniffer Villacís Casa.

## CONFLICT OF INTERESTS

The authors declare that there are no conflict of interests.

## AUTHOR CONTRIBUTIONS

Jimmy Martin‐Delgado and José Joaquin Mira designed the study. Carlos Solís, Lorena Gómez and Gustavo Ramirez Amat were involved in participant recruitment. Jimmy Martin‐Delgado, Mercedes Guilabert and José Joaquin Mira conducted the qualitative study. Jimmy Martin‐Delgado, José Joaquin Mira and Aurora Mula conducted data analysis. All authors reviewed and approved the final draft. The EPD Research Group conformed by Andrea Aguilar‐Aviles, María Castro García, Yamil Cornejo Guerrero, Sandra Gonzalez Chamorro, Lucia Gutierrez Dávila, Karla Mayorga Alvarado, Doménica Mendoza Macías, Carla Mina Curay, Carmita Pérez López, Martha Quijije, Katterin Romero Bello, Daniel Sornoza Arias, Eric Urquizo Rodriguez, Richard Vaca Maridueña, Jenniffer Villacís Casa participated in data collection in each of the participating health centres during the validation study.

## ETHICS STATEMENT

This study was approved by the Research Ethics Committee of Kennedy Clinic Hospital, an approved ethics committee by the Ministry of Public Health in Ecuador (Reference number: HCK‐CEISH‐19‐0041), and the Investigation and Innovation provided a sponsorship letter in Integral Health Institute of the Catholic University of Guayaquil (ISAIN‐2019‐236). Before the focus group or interview, a written informed consent form was obtained from all patients who wished to continue in the study. Participation in this project was entirely voluntary, and participants were informed that they could withdraw from the study at any point without having to provide a reason.

## Supporting information

Supporting information.

Supporting information.

Supporting information.

Supporting information.

Supporting information.

## Data Availability

Data are available from the corresponding author upon reasonable request.
