# Supplementary material for: Development and validation in Ecuador of the EPD Questionnaire, a diabetes‐specific patient‐reported experience and outcome measure: A mixed‐methods study
Source: Health Expect. 2021 Sep 28;25(5):2134–46. doi: 10.1111/hex.13366 (PMC9615093; doi:10.1111/hex.13366)
Supplement: Supplementary file 1 — Supporting information. [file HEX-25--s003.docx]

Supplementary file 1. Interview guide.

Life experience of the diabetic patient

Ecuador.

Patient script

Presentation of objectives and attendees

Question 1: How does diabetes threaten, challenge, exceed or damage my living conditions and my ability to lead a **normal life** with my partner, family, and family?

- Cluster questions:
  - Healthy habits.
  - Symptoms.
  - Autonomy.
  - Faith/Religiosity.
  - Economic impact.
  - Leisure.
  - Goals in life.
  - Physical consequences.
  - Emotional consequences.
  - Couple life and sexuality.

Second question: How does diabetes threaten, challenge, exceed or damage my living conditions and my ability to lead a **normal life** with my friends and at work?

- Cluster questions:
  - Outings away from home.
  - Parties.
  - Professional development opportunities.
  - Limitations in work capacity.
