# Supplementary material for: Development and validation in Ecuador of the EPD Questionnaire, a diabetes‐specific patient‐reported experience and outcome measure: A mixed‐methods study
Source: Health Expect. 2021 Sep 28;25(5):2134–46. doi: 10.1111/hex.13366 (PMC9615093; doi:10.1111/hex.13366)
Supplement: Supplementary file 2 — Supporting information. [file HEX-25--s004.docx]

Supplementary file 2. Translated verbatims of focus groups and semi structured interviews from item development phase.

**Codification**

Female: F / Male: M

Urbal: U / Rural: R

Age: number (nn)

Example. MU64: Male 64 years old of an urban area

| **SIGNS AND SYMPTOMS OF DIABETES** | |
| --- | --- |
| Presence of thirst | *MU53 "I started out very thirsty, so I drank water (...)"*  *MU47 "Even if I drank water, I was thirsty. I was even more thirsty at night and dawn and used the bathroom a lot".* |
| Fatigue | *FR64 "I'm very sleepy"*  *MR51 "when you eat briefly, it gives you decay".*  *FU49 "I feel very tired, as if I have walked for miles."* |
| Hyperglycemia or Hypoglycemia | *FU 57"I'm afraid of traveling, that my glucose levels will go up or down during the trip (...) I use insulin".* |
| **FIRST AND SUBSEQUENT CONSULTATIONS** | |
| Recognition of the disease | *MR53 "I almost had a heart attack, because they tell you straight away that there is no cure".*  *FU57 "my situation was desperate, I just felt like crying."*  *MR47 "when you get the news, it's quite a comedown."*  *MR47 "when I arrived at the health center they told me ´diabetics aside´."*  *MR51 "my wife didn't pay attention to the disease, and she says she's doing fine"*  *MR53 "I am aware that I have the disease"*  *FU50 "she still does not accept her illness"*  *FU49"I had a hard time accepting that I have diabetes."* |
| **COMPLICATIONS OF THE DISEASE** | |
| Nephropathy | *FU44 "what worries me most is getting to dialysis, but there are times when I forget about it."* |
| Diabetic retinopathy | *FR50 "what affected me most about the news, was that I may go blind."*  *MU51 "my fear was to go blind, because you see people who go blind (...) being disabled is worrying".*  *MR53 "to have my eyes blurred."*  *FU61 "vision is what worries me the most."* |
| **LIMITATIONS IN DAILY LIFE** | |
| Work | *MR47 "the economic impact has been more because I don't have the same energy as before to work (...) you get on the side of the road and fall asleep".*  *MR53 "the impact is because we work less (he is a driver) because of your physical condition which makes you tired or sleepy".*  *FR63 "When it wants to happen to me, I think that I am not going to let myself win and I start working harder, it must be because I am not sitting down".*  *FU49 "my children no longer let me work since I got diabetes."*  *MR53 "I don't work like I did when I was young but I still have the energy."*  *"because of diabetes I have never stopped"* |
| Insulin dependence | *MU47 "insulin must always be refrigerated."*  *"it will always be an impediment to anything, having to carry insulin, remedies (...)"*  *FU49 "I don't like to carry my insulin, I just leave it at home (...) if I feel sick I drink water".*  *FU57 "they told me I had to take insulin because my blood sugar wouldn't go down."* |
| Family/social | *MR49 "there is a family impact, an environment that is dispersed, because she doesn't want to follow the diet and she gets angry"*  *MU47 "sometimes people don't understand that you need a hypocaloric diet."*  *MR49 "I eat and drink what I can, and I tell them no more and if they insist I'm out."*  *MU53 "the support depends on how your family behaves, we all have the same diet, but sometimes they tell me they are going out to eat."*  *FU49 "my husband always tells me, to exercise, to eat healthy."* |
| Loss of interest | *MR53 "One is disappointed, there is no longer any interest in buying anything, why buy (...)"*  *MR55 "God takes care of me, he is the only one and me".*  *FU49 "I don't like having to prick my fingers, or take insulin (...) I don't like it (...) because of the pain and sometimes I get blood."*  *FU57 "It is frustrating, to take the test (capillary glucose) and that it is elevated, because I don't know what else to do".* |
| Negative emotions (sadness, anger) | *MR53 "when they ask me something related to the disease it bothers me."*  *FU55 "there are times when my daughters, they tell me not to eat that and I feel bad, because I can't eat it."*  *FU59 "there are times when my husband cooks, and I have to tell him that I can't eat everything he prepares, and he gets angry".*  *FU51 "when my daughters want to forbid me something, it bothers me".*  *FR44"I was feeling down, I felt bad because I had diabetes."*  *FU48 "sometimes out of the blue I cry, I feel sad (...) if my husband tells me something he doesn't like I just cry."* |
| Constant fear that something bad might happen | *FR61 "there is a fear that something might happen."*  *MR62 "(...) they took me to the hospital at night, I was afraid, not to death. I told my wife that tomorrow I will give her my will with the attorney of everything, that's what I was afraid of, I'm afraid that everything will be thrown away."*  *FU48 "What worries me most is that something happens and I have to have an amputation".*  *MU63 "what worries me the most is that she doesn't take care of herself."*  *MU63 "since I work in a hospital every time I hear about a patient going blind or having an amputation I think of her, and I worry a lot."*  *FU49 "I'm worried, having a stroke and leaving my children behind."* |
| **CONTROL OF RISK FACTORS AND THERAPEUTIC REGIMEN** | |
| Healthy habits | *MR47 "you get angry about habits".*  *MR68 "stop drinking (alcoholic beverages)."*  *FU51 "diet is something that worries me about diabetes because I do it for a few days, but then I get tired."*  *MU51 "the hardest thing is to get used to eating healthy".*  *FU52 "when my glycemic was really bad, I prepared my own food".*  *FU52 "when there is no time it is difficult to fulfill it (healthy habits)".*  *MU49 "I almost never do the exercises, because there is no time."*  *FR51 "the most complicated thing for me has been the habits (...) but really for me the problem has been the food."*  *FU48 "for me it has been impossible, because we eat at my brother's place, nobody has diabetes at his house, so I can only eat small portions but not diet".* |
| Health education | *MR51 "the (patient with diabetes) group doctor gives us the lectures (education)".*  *FU48 "In the group they tell us how to feed ourselves, what to eat, how to administer insulin".*  *FU52 "with the endocrinologist or the doctor at the center."*  *MU49 "self-instructing"*  *FU58 "through my children, I asked them to look on the internet (...) but I got scared."*  *FR62"They didn't explain to me, the doctor just said I had high blood sugar and that's all.*  *MU63 "The doctor did explain to me, he told me that I had to change the food, how to cook it (...)".*  *FU54 "only what the doctor can tell you in the office".*  *FU49 "here at the social security, they give us talks."*  *FU48 "I was stabilized by a private doctor and a nutritionist (...) they explained to me."* |
| Nutritional education | *FU49 "sometimes I cook myself, (...) now I'm forbidden to drink coke".*  *FU64 "I already gave myself to grief, to eat like this".*  *FR44 "I do eat everything, but not in excess, only by small portions".*  *MR62 "It is hard to change our diet at home and even socially if we are invited to a party, or they give us something sweet and we say no, they think it is out of contempt and they don't understand that it is for our health, sometimes they insult us.”*  *MR49 "I don't have diabetes but my wife does (...) and the truth is that she doesn't adapt to the diet, I try".*  *FR55"a very big problem was the food, because they had to cook me apart, and they didn't feel well and neither did I."*  *FR55 "in the groups they do give talks, but I have stopped coming."*  *FU63 "the doctor who gives us the consultation explains that we can't eat any sweets."*  *FU48 "The most difficult thing at the beginning was the food (...) but one day a doctor explained to me how I should eat.* |
| Socioeconomic status | *FU52 "sometimes we had to wait to buy the medicines (privately), because there was no money, but now my husband makes the effort to pay the social security."*  *FU48 "the test strips are the most expensive thing, sometimes I try".*  *FU48 "I have to buy my own medicine and insulin pens."*  *FU48 "The medicine has to be taken perennially (...) and it costs me almost $40 a box and it's a box every 15 days (...) the insulin pen is only $30 a month, so I prefer it".* |
| Commitment to the therapeutic regimen | *FU55 "I was told that the disease is bearable if you are conscious about medication and nutrition."*  *FR64 "walking, exercise, all of that is difficult."*  *FU48 "when I travel I forget that I have diabetes".*  *FR64 "I have to die of something" (in reference to being told that he should not eat some food).*  *FU54 "I tell them to let me eat, we are all going to die, but I'm going to eat."*  *FU50 "they always tell me, don't eat this but can you imagine, going to a birthday party and not eating cake."*  *MU51 "I currently use the insulin pen, previously only pills."* |
| Alternative treatments | *FU59"sometimes the desperation of the disease makes you run to different parts and also when someone else tells you something else different."*  *FR75 "I didn't take the medicines, I opted for natural medicine but now I have been taking the medicines for about 7 years."*  *MU49 "I don't take medicines, I drink natural waters".* |
| Erroneous beliefs | *FR55"the bad thing about insulin is that it makes you blind."*  *FR44 "I told the doctor that the pills here are not good for me."*  *FR64 "the doctors told me not to stop taking insulin, but I stopped anyway".* |
