# Supplementary material for: Development and validation in Ecuador of the EPD Questionnaire, a diabetes‐specific patient‐reported experience and outcome measure: A mixed‐methods study
Source: Health Expect. 2021 Sep 28;25(5):2134–46. doi: 10.1111/hex.13366 (PMC9615093; doi:10.1111/hex.13366)
Supplement: Supplementary file 3 — Supporting information. [file HEX-25--s001.docx]

**Código: _______________ Población: Urbana_____ Rural _____**

| Las siguientes preguntas buscan averiguar sobre su experiencia con la atención que ha recibido de parte del doctor, la enfermera, la auxiliar y cualquier otro servicio o profesional de salud en el diagnóstico, manejo y tratamiento de la diabetes. Marque por favor con una X en el círculo más cercano a su realidad. Siempre con respecto al último mes. | | | |
| --- | --- | --- | --- |
|  | | Raras veces Algunos días Casi siempre Siempre | |
| 1. He podido conversar con el doctor acerca de que es importante para mí. | | 1 2 3 4 | |
| 2. El doctor me ha explicado lo que puedo comer. | | 1 2 3 4 | |
| 3. He recibido información acerca del ejercicio que puedo hacer. | | 1 2 3 4 | |
| 4. He sentido que el doctor me escuchó en la consulta. | | 1 2 3 4 | |
| 5. He recibido información con palabras que pude entender. | | 1 2 3 4 | |
| 6. He aprendido a sobrellevar mi diabetes. | | 1 2 3 4 | |
| 7. Estoy preparado para saber qué hacer en caso de que ocurra algo inesperado con mi diabetes. | | 1 2 3 4 | |
| 8. Puedo contactar con mi doctor siempre que lo necesito. | | 1 2 3 4 | |
| **Las siguientes preguntas buscan averiguar sobre sus emociones y como es su experiencia de vivir con diabetes. Marque por favor con una X en el círculo más cercano a su realidad. Siempre con respecto al último mes.** | | |  |
|  | Raras veces Algunos días Casi siempre Siempre | |  |
| 1. Tengo mucha sed ahora último, así tome agua. | 4 3 2 1 | |  |
| 2. Tengo decaimiento. | 4 3 2 1 | |  |
| 3. Tengo miedo de quedar ciego. | 4 3 2 1 | |  |
| 4. Tengo miedo de llegar a diálisis. | 4 3 2 1 | |  |
| 5. He abandonado el tratamiento de la diabetes porque tengo dificultad para poder pagarlo. | 4 3 2 1 | |  |
| 6. Tengo problemas para cumplir con mi trabajo. | 4 3 2 1 | |  |
| 7. He estado solo frente a la enfermedad. | 4 3 2 1 | |  |
| 8. He tenido problemas con mi familia o amigos por la diabetes (por ejemplo, alguna discusión sobre lo que puedo comer). | 4 3 2 1 | |  |
| 9. He dejado de salir de vacaciones o de fin de semana por el tratamiento con la diabetes. | 4 3 2 1 | |  |
| 10. Uso tratamientos naturales en lugar de las pastillas. | 4 3 2 1 | |  |
| 11. Tengo problemas para saber cuánto debo comer. | 4 3 2 1 | |  |
| 12. Me he sentido vencido por vivir con diabetes. | 4 3 2 1 | |  |

**English Translation**

**Code: _______________ Town: Urban_____ Rural _____**

| The following questions seek to find out about your experience with the care you have received from your doctor, nurse, nurse's aide and any other health service or professional in the diagnosis, management and treatment of diabetes. Please mark with an X in the circle closest to your reality. Always with respect to the last month. | | | |
| --- | --- | --- | --- |
|  | | Hardly ever Someday Almost everyday Eveyday | |
| 1. I have been able to talk to the doctor about what is important to me. | | 1 2 3 4 | |
| 2. The doctor has explained to me what I can eat. | | 1 2 3 4 | |
| 3. I have received information about the exercise I can do. | | 1 2 3 4 | |
| 4. I felt that the doctor listened to me in the consultation. | | 1 2 3 4 | |
| 5. I have received information in words that I could understand. | | 1 2 3 4 | |
| 6. I have learned to cope with my diabetes. | | 1 2 3 4 | |
| 7. I am prepared to know what to do in case something unexpected happens with my diabetes. | | 1 2 3 4 | |
| 8. I can contact my doctor whenever I need to. | | 1 2 3 4 | |
| **The following questions seek to find out about your emotions and what your experience of living with diabetes is like. Please mark with an X in the circle closest to your reality. Always with respect to the last month.** | | |  |
|  | Hardly ever Someday Almost everyday Eveyday | |  |
| 1. I am very thirsty even if I drink water. | 4 3 2 1 | |  |
| 2. I have been feeling weak | 4 3 2 1 | |  |
| 3. I am afraid I'll go blind | 4 3 2 1 | |  |
| 4. I am afraid to go to dialysis. | 4 3 2 1 | |  |
| 5. I have stopped treatment for diabetes because I have difficulty paying for it. | 4 3 2 1 | |  |
| 6. I have trouble getting my work done. | 4 3 2 1 | |  |
| 7. I have been alone against my illness. | 4 3 2 1 | |  |
| 8. I have had problems with my family or friends because of diabetes (e.g., an argument about what I can eat). | 4 3 2 1 | |  |
| 9. I have stopped going on vacations or weekends because of my diabetes treatment. | 4 3 2 1 | |  |
| 10. I use natural treatments instead of pills. | 4 3 2 1 | |  |
| 11. I have trouble knowing how much to eat. | 4 3 2 1 | |  |
| 12. I have felt defeated by living with diabetes. | 4 3 2 1 | |  |
