# Supplementary material for: Development and validation in Ecuador of the EPD Questionnaire, a diabetes‐specific patient‐reported experience and outcome measure: A mixed‐methods study
Source: Health Expect. 2021 Sep 28;25(5):2134–46. doi: 10.1111/hex.13366 (PMC9615093; doi:10.1111/hex.13366)
Supplement: Supplementary file 4 — Supporting information. [file HEX-25--s005.docx]

Supplementary file 4.

Table 1. Patient reported experience results related with years of diagnosis.

|  | Years of diagnosis  (N=480) | Mean ± SD | P-value* |
| --- | --- | --- | --- |
| 1. I have been able to talk to the doctor about what is important to me. | ≤1 (n=66) | 2.6 ± 1 | 0.2 |
|  | 2-10 (n=271) | 2.8 ± 0.9 |  |
|  | 11-20 (n=108) | 2.9 ± 1 |  |
|  | > 20 (n=35) | 2.9 ± 1 |  |
| 2. The doctor has explained to me what I can eat. | ≤1 (n=66) | 2.3 ± 0.8 | 0.06 |
|  | 2-10 (n=271) | 2.5 ± 1 |  |
|  | 11-20 (n=108) | 2.6 ± 1 |  |
|  | > 20 (n=35) | 2.9 ± 1 |  |
| 3. I have received information about the exercise I can do. | ≤1 (n=66) | 1.6 ± 0.7 | <0.001 |
|  | 2-10 (n=271) | 2.1 ± 1 |  |
|  | 11-20 (n=108) | 2.2 ± 1.1 |  |
|  | > 20 (n=35) | 2.5 ± 1.1 |  |
| 4. I felt that the doctor listened to me in the consultation. | ≤1 (n=66) | 2.4 ± 0.8 | 0.001 |
|  | 2-10 (n=271) | 2.4 ± 1 |  |
|  | 11-20 (n=108) | 2.6 ± 1 |  |
|  | > 20 (n=35) | 3.1 ± 0.8 |  |
| 5. I have received information in words that I could understand. | ≤1 (n=66) | 1.7 ± 0.8 | <0.001 |
|  | 2-10 (n=271) | 2.2 ± 1 |  |
|  | 11-20 (n=108) | 2.3 ± 1.1 |  |
|  | > 20 (n=35) | 2.8 ± 1.1 |  |
|  | 11-20 (n=108) | 3.0 ± 0.1 |  |
|  | > 20 (n=35) | 2.8 ± 0.1 |  |
| 6. I have learned to cope with my diabetes. | ≤1 (n=66) | 1.7 ± 0.8 | <0.001 |
|  | 2-10 (n=271) | 2.1 ± 0.9 |  |
|  | 11-20 (n=108) | 2.1 ± 1 |  |
|  | > 20 (n=35) | 2.8 ± 1 |  |
| 7. I am prepared to know what to do in case something unexpected happens with my diabetes. | ≤1 (n=66) | 2.4 ± 0.8 | 0.2 |
|  | 2-10 (n=271) | 2.4 ± 0.9 |  |
|  | 11-20 (n=108) | 2.5 ± 0.9 |  |
|  | > 20 (n=35) | 2.7 ± 0.9 |  |
| 8. I can contact my doctor whenever I need to. | ≤1 (n=66) | 2.5 ± 0.9 | 0.1 |
|  | 2-10 (n=271) | 2.6 ± 1 |  |
|  | 11-20 (n=108) | 2.7 ± 1 |  |
|  | > 20 (n=35) | 2.2 ± 0.9 |  |
| Factor 1: 2, 4, 7 | ≤1 (n=66) | 5.0 ± 2.2 | 0.005 |
|  | 2-10 (n=271) | 6.4 ± 2.7 |  |
|  | 11-20 (n=108) | 6.5 ± 3.1 |  |
|  | > 20 (n=35) | 8.1 ± 2.8 |  |
| Factor 2: 3, 5, 6 | ≤1 (n=66) | 7.1 ± 2.4 | <0.001 |
|  | 2-10 (n=271) | 7.3 ± 2.6 |  |
|  | 11-20 (n=108) | 7.6 ± 2.7 |  |
|  | > 20 (n=35) | 8.7 ± 2.0 |  |
| Factor 3: 1, 8 | ≤1 (n=66) | 5.1 ± 1.9 | 0.2 |
|  | 2-10 (n=271) | 5.4 ± 1.8 |  |
|  | 11-20 (n=108) | 5.5 ± 1.8 |  |
|  | > 20 (n=35) | 5.1 ± 1.6 |  |
| Total | ≤1 (n=66) | 17.2 ± 4.3 | <0.001 |
|  | 2-10 (n=271) | 19.0 ± 5.4 |  |
|  | 11-20 (n=108) | 19.6 ± 6.1 |  |
|  | > 20 (n=35) | 21.8 ± 5.1 |  |

Kruskal-Wallis was used.

Table 2. Patient reported outcomes related with years of diagnosis.

|  | Years of diagnosis (N=480) | Mean ± SD | P-value* |
| --- | --- | --- | --- |
| 1. I am very thirsty even if I drink water. | ≤1 (n=66) | 3.4 ± 0.6 | 0.05 |
|  | 2-10 (n=271) | 3.2 ± 0.7 |  |
|  | 11-20 (n=108) | 3.3 ± 0.8 |  |
|  | > 20 (n=35) | 3.0 ± 0.9 |  |
| 2. I have been feeling weak. | ≤1 (n=66) | 3.4 ± 0.6 | 0.06 |
|  | 2-10 (n=271) | 3.3 ± 0.6 |  |
|  | 11-20 (n=108) | 3.4 ± 0.7 |  |
|  | > 20 (n=35) | 3.1 ± 0.8 |  |
| 3. I am afraid I'll go blind | ≤1 (n=66) | 2.9 ± 0.8 | <0.001 |
|  | 2-10 (n=271) | 2.6 ± 1 |  |
|  | 11-20 (n=108) | 2.6 ± 1 |  |
|  | > 20 (n=35) | 1.8 ± 1 |  |
| 4. I am afraid to go to dialysis. | ≤1 (n=66) | 3.3 ± 0.8 | <0.001 |
|  | 2-10 (n=271) | 2.9 ± 1 |  |
|  | 11-20 (n=108) | 2.9 ± 1.1 |  |
|  | > 20 (n=35) | 1.8 ± 1 |  |
| 5. I have stopped treatment for diabetes because I have difficulty paying for it. | ≤1 (n=66) | 3.3 ± 0.5 | 0.02 |
|  | 2-10 (n=271) | 3.4 ± 0.6 |  |
|  | 11-20 (n=108) | 3.6 ± 0.5 |  |
|  | > 20 (n=35) | 3.5 ± 0.6 |  |
| 6. I have trouble getting my work done. | ≤1 (n=66) | 3.3 ± 0.6 | 0.1 |
|  | 2-10 (n=271) | 3.5 ± 0.5 |  |
|  | 11-20 (n=108) | 3.5 ± 0.6 |  |
|  | > 20 (n=35) | 3.3 ± 0.8 |  |
| 7.I have been alone in the face of illness. | ≤1 (n=66) | 3.3 ± 0.6 | 0.3 |
|  | 2-10 (n=271) | 3.4 ± 0.7 |  |
|  | 11-20 (n=108) | 3.4 ± 0.7 |  |
|  | > 20 (n=35) | 3.4 ± 0.7 |  |
| 8. I have had problems with my family or friends because of diabetes (for example, an argument about what I can eat). | ≤1 (n=66) | 3.5 ± 0.6 | 0.02 |
|  | 2-10 (n=271) | 3.3 ± 0.6 |  |
|  | 11-20 (n=108) | 3.4 ± 0.6 |  |
|  | > 20 (n=35) | 3.6 ± 0.6 |  |
| 9. I have stopped going on vacations or weekends because of my diabetes treatment. | ≤1 (n=66) | 3.3 ± 0.5 | 0.2 |
|  | 2-10 (n=271) | 3.4 ± 0.6 |  |
|  | 11-20 (n=108) | 3.5 ± 0.6 |  |
|  | > 20 (n=35) | 3.4 ± 0.8 |  |
| 10. I use natural treatments instead of pills. | ≤1 (n=66) | 3.4 ± 0.5 | 0.1 |
|  | 2-10 (n=271) | 3.5 ± 0.5 |  |
|  | 11-20 (n=108) | 3.6 ± 0.6 |  |
|  | > 20 (n=35) | 3.5 ± 0.8 |  |
| 11. I have trouble knowing how much to eat. | ≤1 (n=66) | 3.5 ± 0.6 | 0.002 |
|  | 2-10 (n=271) | 3.3 ± 0.7 |  |
|  | 11-20 (n=108) | 3.4 ± 0.7 |  |
|  | > 20 (n=35) | 2.9 ± 0.8 |  |
| 12. I have felt defeated by living with diabetes. | ≤1 (n=66) | 3.5 ± 0.6 | 0.3 |
|  | 2-10 (n=271) | 3.4 ± 0.6 |  |
|  | 11-20 (n=108) | 3.4 ± 0.7 |  |
|  | > 20 (n=35) | 3.2 ± 0.8 |  |
| Factor 1: 1, 2, 8, 11, 12 | ≤1 (n=66) | 17.3 ± 2.8 | 0.02 |
|  | 2-10 (n=271) | 16.5 ± 2.7 |  |
|  | 11-20 (n=108) | 16.9 ± 2.9 |  |
|  | > 20 (n=35) | 15.6 ± 2.8 |  |
| Factor 2: 3, 4 | ≤1 (n=66) | 6.2 ± 1.4 | <0.001 |
|  | 2-10 (n=271) | 5.5 ± 1.9 |  |
|  | 11-20 (n=108) | 5.6 ± 2.0 |  |
|  | > 20 (n=35) | 3.6 ± 2.0 |  |
| Factor 3: 5, 6, 7, 9, 10 | ≤1 (n=66) | 16.6 ± 2.6 | 0.2 |
|  | 2-10 (n=271) | 17.2 ± 2.4 |  |
|  | 11-20 (n=108) | 17.5 ± 2.3 |  |
|  | > 20 (n=35) | 17.1 ± 2.4 |  |
| Total | ≤1 (n=66) | 40.1 ± 4.6 | <0.001 |
|  | 2-10 (n=271) | 39.2 ± 4.6 |  |
|  | 11-20 (n=108) | 39.9 ± 4.9 |  |
|  | > 20 (n=35) | 36.2 ± 4.8 |  |

Kruskal-Wallis was used.
