# Supplementary material for: Development and validation in Ecuador of the EPD Questionnaire, a diabetes‐specific patient‐reported experience and outcome measure: A mixed‐methods study
Source: Health Expect. 2021 Sep 28;25(5):2134–46. doi: 10.1111/hex.13366 (PMC9615093; doi:10.1111/hex.13366)
Supplement: Supplementary file 5 — Supporting information. [file HEX-25--s002.docx]

Supplementary file 5. Minimal Clinical Change of the EPD Questionnaire

| Scale identification | Mean ± SD  All patients (N= 488) | Mean ± SD  Urban patients (N= 281) | Alpha coefficient | 1.0 SEM | 0.5 SES | 0.5 RS |
| --- | --- | --- | --- | --- | --- | --- |
| Total PREM | 19.3 ± 5.6 | 19,8 ± 6,2 | 0.87 | 2.46 | 2.8 | 3.1 |
| Factor 1: Information | 6.4 ± 2.8 | 6,6 ± 3,2 | 0.93 | 0.74 | 1.4 | 1.6 |
| Factor 2: Patient centered care | 7.5 ± 2.6 | 7,7 ± 2,8 | 0.92 | 0.73 | 1.3 | 1.4 |
| Factor 3: Care delivery | 5.4 ± 1.8 | 5,6 ± 1,8 | 0.83 | 0.74 | 0.9 | 0.9 |
| Total PROM | 39.2 ± 5 | 40,2 ± 4,9 | 0.83 | 2.18 | 2.5 | 2.4 |
| Factor 1: Symptoms and burnout | 16.6 ± 2.8 | 17,1 ± 2,7 | 0.88 | 0.97 | 1.4 | 1.4 |
| Factor 2: Worries and fears | 5.4 ± 1.9 | 5,7 ± 1,8 | 0.81 | 0.83 | 1 | 0.9 |
| Factor 3: Social limitations | 17.2 ± 2.5 | 17,4 ± 2,5 | 0.88 | 0.87 | 1.3 | 1.3 |
